# Patterns of infection and day care utilization and risk of childhood acute lymphoblastic leukaemia

**DOI:** 10.1054/bjoc.1999.0905

**Published:** 1999-12-08

**Authors:** J P Neglia, M S Linet, X O Shu, R K Severson, J D Potter, A C Mertens, W Wen, J H Kersey, L L Robison

**Affiliations:** Departments of PediatricsLaboratory Medicine and Pathology, University of Minnesota School of Medicine, Minneapolis, MN, USA; Division of Epidemiology, University of Minnesota School of Public Health, Minneapolis, MN, USA; Division of Cancer Epidemiology and Genetics, National Cancer Institute, Bethesda, MD, USA; Department of Pediatrics, University of South Carolina School of Medicine, Columbia, SC, USA; Fred Hutchinson Cancer Research Center, Seattle, WA, USA; Karmanos Cancer Institute, Detroit, MI, USA

**Keywords:** childhood acute lymphoblastic leukaemia, infections, day care

## Abstract

To investigate if decreased exposure to common childhood infections is associated with risk of childhood acute lymphoblastic leukaemia (ALL) we conducted a case–control study of 1842 newly diagnosed and immunophenotypically defined cases of ALL under age 15, and 1986 matched controls in the US. Data regarding day care, sibship size and common childhood infections were obtained through parental interviews. Data were analysed stratified by leukaemia lineage and separately for ‘common’ childhood ALL (age 2–5 years, CD19, CD10-positive). Neither attendance at day care nor time at day care was associated with risk of ALL overall or ‘common’ ALL. Ear infections during infancy were less common among cases, with odds ratios of 0.86, 0.83, 0.71 and 0.69 for 1, 2–4, 5+ episodes, and continuous infections respectively (trend *P* = 0.026). No effect of sibship size or birth interval was seen. With one exception (ear infections), these data do not support the hypothesis that a decrease in the occurrence of common childhood infection increases risk of ALL. © 2000 Cancer Research Campaign


*British Journal of Cancer* (2000) **82**(1), 234–240
© 2000 Cancer Research Campaign
Article no. bjoc.1999.0905

### Patterns of infection and day care utilization and risk of childhood acute lymphoblastic leukaemia

**JP Neglia[1,3], MS Linet[4], XO Shu[5], RK Severson[7], JD Potter[6], AC Mertens[1,3], W Wen[5], JH Kersey[1,2] and LL Robison[1,3]**

Departments of [1]Pediatrics and [2]Laboratory Medicine and Pathology, University of Minnesota School of Medicine, Minneapolis, MN, USA; [3]Division of Epidemiology, University of Minnesota School of Public Health, Minneapolis, MN, USA; [4]Division of Cancer Epidemiology and Genetics, National Cancer Institute, Bethesda, MD, USA; [5]Department of Pediatrics, University of South Carolina School of Medicine, Columbia, SC, USA; [6]Fred Hutchinson Cancer Research Center, Seattle, WA, USA; [7]Karmanos Cancer Institute, Detroit, MI, USA

**Summary** To investigate if decreased exposure to common childhood infections is associated with risk of childhood acute lymphoblastic leukaemia (ALL) we conducted a case–control study of 1842 newly diagnosed and immunophenotypically defined cases of ALL under age 15, and 1986 matched controls in the US. Data regarding day care, sibship size and common childhood infections were obtained through parental interviews. Data were analysed stratified by leukaemia lineage and separately for ‘common’ childhood ALL (age 2–5 years, CD19, CD10-positive). Neither attendance at day care nor time at day care was associated with risk of ALL overall or ‘common’ ALL. Ear infections during infancy were less common among cases, with odds ratios of 0.86, 0.83, 0.71 and 0.69 for 1, 2–4, 5+ episodes, and continuous infections respectively (trend $P = 0.026$). No effect of sibship size or birth interval was seen. With one exception (ear infections), these data do not support the hypothesis that a decrease in the occurrence of common childhood infection increases risk of ALL. © 2000 Cancer Research Campaign

**Keywords**: childhood acute lymphoblastic leukaemia; infections; day care

Acute lymphoblastic leukaemia (ALL), the most common childhood cancer worldwide, occurs at annual rates of between three and four cases per 100 000 children under age 15 in most developed countries (Gurney, 1995; Parkin et al, 1998). Apart from genetic disorders and ionizing radiation, there are no clear aetiological risk factors for ALL (Ross et al, 1994; Pui, 1995; Greaves, 1997; Sandler and Ross, 1997). Investigations of more ubiquitous exposures, including low frequency electromagnetic radiation, have been inconsistent (Savitz et al, 1988; Feychting and Ahlbom, 1993; Linet et al, 1997). ALL is a heterogeneous disorder; however, the majority of cases arise from cells which express CD10 and CD19 cell-surface proteins consistent with B-lineage differentiation (Pui et al, 1993; Kersey, 1997). This phenotype is most common among children diagnosed with ALL between ages 2 and 5, corresponding to the observed age-specific incidence peak (Gurney, 1995; Sandler and Ross, 1997). Greaves hypothesized that the pathogenesis of ‘common’ B-lineage childhood ALL is a two-step process (Greaves, 1988). In the first step, the developmentally driven proliferation of B-precursor cells results in spontaneous mutation of a small population of cells. In the second event, additional mutation or proliferation of the initially mutated cells results from infectious exposures. In this model, *delayed* exposure to common childhood infections leads to a leukaemic outcome in a small number of children as a result of stimulation of an expanded population of pre-leukaemic cells by virtue of later infection. International and regional patterns of ALL incidence demonstrating lower rates in developing countries support this hypothesis (Greaves and Chan, 1986; Greaves, 1988, 1993).

In a large case–control study, the Children’s Cancer Group (CCG) and the National Cancer Institute investigated patterns of infection, surrogates of infection and risk of childhood ALL within immunophenotypic subgroups. We hypothesized that children with ALL would have had fewer childhood infections or have been less likely to be in high-exposure environments (i.e. day care) than controls.

### METHODS

### Case ascertainment

Cases of newly diagnosed ALL in children under 15 years of age were ascertained from CCG member institutions throughout the USA. To be eligible, cases had to be newly diagnosed with ALL between 1 January 1989 and 15 June 1993, and the biological mother had to be available for interview, speak English and have a telephone in the household. Submission of a diagnostic bone marrow specimen for immunophenotyping was required. A total of 2081 eligible cases were identified during the study period and a telephone interview completed for 1914 (92.1% of eligible cases). The reasons for non-participation of 167 eligible cases are shown in Table 1.

### Control selection

Controls were randomly selected using a random digit-dialling methodology previously described (Robison and Daigle, 1984) and individually matched to cases on age (within 25% of age at diagnosis of the case, with a maximum difference of ± 2 years), race (white, black, or other, although this match was relaxed if a

*Received 5 March 1999*
*Revised 1 June 1999*
*Accepted 14 June 1999*

*Correspondence to*: JP Neglia, Children’s Cancer Group, PO Box 60012, Arcadia, CA 91066-6012, USA

**Table 1** Characteristics of cases and controls

| | Cases | Controls | *P*-value |
|---|---|---|---|
| Maternal interview | | | |
| Eligible | 2081 | 2597 | |
| Completed interview | 1914 (92.1%) | 1987 (76.5%) | |
| Matched set | 1842 (88.6%) | 1986 (76.5%) | |
| Non-response | 167 (7.9%) | 610 (23.5%) | |
| Physician refusal | 41 (2.0%) | – | |
| Parental refusal | 70 (3.4%) | 457 (17.6%) | |
| Lost to follow-up | 18 (0.9%) | 17 (0.7%) | |
| Other | 38 (1.8%) | 136 (5.2%) | |
| Gender – male | 1018 (55.2%) | 1076 (54.2%) | 0.54 |
| Age | | | |
| <12 months | 64 (3.5%) | 81 (4.1%) | 0.07 |
| 12–23 months | 138 (7.5%) | 189 (9.5%) | |
| 2–5 years | 1020 (55.4%) | 1038 (52.3%) | |
| 6–10 years | 408 (22.2%) | 466 (23.5%) | |
| 11–15 years | 212 (11.5%) | 212 (10.7%) | |
| Race | | | |
| White | 1492 (81.0%) | 1720 (86.6%) | <0.01 |
| Black | 109 (5.9%) | 94 (4.7%) | |
| Hispanic | 153 (8.3%) | 121 (6.1%) | |
| Other | 88 (4.8%) | 51 (2.6%) | |
| Maternal education | | | |
| = High school | 797 (43.3%) | 762 (38.4%) | <0.01 |
| Some post high school | 592 (32.1%) | 701 (35.3%) | |
| College + | 453 (24.6%) | 523 (26.3%) | |
| Paternal education[a] | | | |
| ≤ High school | 676 (41.8%) | 638 (37.1%) | <0.01 |
| Some post high school | 480 (29.7%) | 510 (29.6%) | |
| College + | 462 (28.6%) | 574 (33.4%) | |
| Income | | | |
| <10 000 | 217 (11.8%) | 176 (8.9%) | <0.01 |
| 10 000–19 999 | 390 (21.2%) | 370 (18.6%) | |
| 20 000–29 999 | 433 (23.5%) | 475 (23.9%) | |
| 30 000–39 999 | 334 (18.1%) | 369 (18.6%) | |
| 40 000–49 999 | 204 (11.1%) | 221 (11.1%) | |
| 50 000+ | 250 (13.6%) | 357 (18.0%) | |
| Unknown | 14 (0.8%) | 18 (0.9%) | |
| Immunophenotype | | | |
| T-cell | 183 (9.9%) | | |
| Early pre-B-cell | 893 (48.5%) | | |
| Pre-B-cell | 233 (12.6%) | | |
| B not specified | 231 (12.5%) | | |
| Unknown | 302 (16.4%) | | |

[a]Based on 1618 cases and 1722 matched controls who responded to paternal interview.

control was not obtainable for a given case) and telephone area code and exchange. T-cell case–controls were also matched for gender. A total of 2597 eligible controls were identified, of whom 1987 (76.5%) participated. One control was excluded because the case was later found to be ineligible for the study. Reasons for non-participation are shown in Table 1. Matched controls were not found for 72 (3.6%) interviewed cases, resulting in 1842 case–control pairs (1704 sets of 1:1 match, 132 sets of 1:2 match and 6 sets of 1:3 match).

### Interview

The structured interview included questions about the subject's and mother's health history, details of pregnancy and birth, childhood illnesses, illnesses of siblings and parental occupation. The maternal interview included details of day care and pre-school attendance (whether or not the index child ever attended day care or pre-school, approximate dates of attendance, and average number of hours per day). Day care or pre-school attendance in the year prior to the ALL diagnosis (cases) or index date (controls) was excluded to eliminate the possibility that symptoms of ALL itself decreased attendance at day care or pre-school. There was considerable overlap in day care and pre-school attendance times, thus total time spent by a child in these activities were pooled for analysis and is referred to as 'day care'.

Mothers of cases and controls residing in a nine-state region of the northeastern USA participated in an additional in-home interview (Kleinerman et al, 1997; Hatch et al, 1998), which included more detailed information on the childhood health history and several common childhood illnesses.

### Immunophenotype determinations

A standard panel of monoclonal antibodies was applied to all diagnostic bone marrow specimens to determine B- or T-lineage. A subset of B-lineage leukaemias was further classified by the determination of cytoplasmic immunoglobulin. Cases were assigned to one of the following mutually exclusive groups: T-cell ALL, early pre-B ALL (B-lineage markers and cytoplasmic immunoglobulin negative), pre-B ALL (B-lineage markers and cytoplasmic immunoglobulin positive), B-lineage ALL – not otherwise specified (NOS) (B-lineage markers but cytoplasmic immunoglobulin not performed), or unclassifiable. 'Common' childhood ALL was defined as those cases determined to be of B-lineage expressing both the CD10 and CD19 surface antigens, and diagnosed in children aged 2–5 years.

### Statistical analysis

Data were analysed for all types of ALL as a group and within strata defined by age or immunophenotypic subtype. Conditional logistic regression was used to calculate odds ratios (OR) and 95% confidence intervals (CI) as an approximation of relative risk with control for potential confounders. Tests for trend were performed by treating levels of categorical data as continuous variables in the logistic model. Analyses were adjusted for maternal education, family income and race.

### RESULTS

### Study participants

The characteristics of 1842 cases and 1986 controls are shown in Table 1. Cases were predominately male, white and between the ages of 2 and 5 years. As compared to the cases, controls were more often white, and were of higher socio-economic status as evidenced by differences in income, and maternal and paternal education (Table 1). Phenotypically, 9.9% of ALL cases were T-lineage, 73.7% B-lineage, and no clear lineage could be assigned in 16.4%. Among 1357 B-lineage cases, 633 (46.6%) were designated 'common' childhood ALL.

### Day care and pre-school (Table 2)

The likelihood of ever attending day care was similar among cases and controls (OR 0.96; 95% CI 0.82–1.12). No inverse association (protective effect) of day care attendance (vs no day care attendance) was seen among children with 'common' ALL (OR 0.96,

*© 2000 Cancer Research Campaign*

**Table 2** Day care[a] and risk of childhood ALL

| | Total ALL | | | 'Common' ALL CD10+, CD19+, Age 2–5 | | | All other ALL | | |
|---|---|---|---|---|---|---|---|---|---|
| | Cases *n* = 1744 | Controls *n* = 1879 | OR (95% CI) | Cases *n* = 633 | Controls *n* = 687 | OR (95% CI) | Cases *n* = 1111 | Controls *n* = 1192 | OR (95% CI) |
| Any day care | | | | | | | | | |
| No | 914 | 949 | 1.00 | 413 | 429 | 1.00 | 501 | 520 | 1.00 |
| Yes | 830 | 930 | 0.96 (0.82–1.12) | 220 | 258 | 0.96 (0.75–1.24) | 610 | 672 | 0.95 (0.78–1.16) |
| Age at start of day care | | | | | | | | | |
| No day care | 914 | 949 | 1.00 | 413 | 429 | 1.00 | 501 | 520 | 1.00 |
| ≤ 6 months | 165 | 201 | 0.91 (0.72–1.15) | 67 | 78 | 1.02 (0.70–1.48) | 98 | 123 | 0.85 (0.62–1.16) |
| 7–12 months | 66 | 76 | 0.99 (0.70–1.41) | 27 | 28 | 1.23 (0.70–2.15) | 39 | 48 | 0.90 (0.57–1.41) |
| 13–24 months | 114 | 113 | 1.06 (0.79–1.41) | 41 | 49 | 0.88 (0.56–1.38) | 73 | 64 | 1.18 (0.80–1.73) |
| > 24 months | 185 | 540 | 0.94 (0.77–1.14) | 85 | 103 | 0.84 (0.57–1.23) | 400 | 437 | 0.96 (0.77–1.21) |
| Test for trend | | | *P* = 0.712 | | | *P* = 0.423 | | | *P* = 0.995 |
| Time in day care (days in day care, regardless how many hours/day) | | | | | | | | | |
| No day care | 914 | 949 | 1.00 | 413 | 429 | 1.00 | 501 | 520 | 1.00 |
| <3 months | 78 | 73 | 1.07 (0.77–1.50) | 33 | 30 | 1.08 (0.64–1.83) | 45 | 43 | 1.05 (0.67–1.62) |
| 3–6 months | 74 | 68 | 1.09 (0.76–1.55) | 35 | 26 | 1.31 (0.75–2.30) | 39 | 42 | 0.92 (0.58–1.48) |
| 7–12 months | 218 | 266 | 0.88 (0.71–1.10) | 47 | 71 | 0.74 (0.48–1.12) | 171 | 195 | 0.93 (0.72–1.22) |
| > 12 months | 460 | 523 | 0.94 (0.78–1.13) | 105 | 131 | 0.96 (0.70–1.32) | 355 | 392 | 0.95 (0.75–1.20) |
| Test for trend | | | *P* = 0.407 | | | *P* = 0.560 | | | *P* = 0.597 |
| Day care before age 2 | | | | | | | | | |
| No | 1399 | 1489 | 1.00 | 498 | 532 | 1.00 | 901 | 957 | 1.00 |
| Yes | 345 | 390 | 0.99 (0.84–1.17) | 135 | 155 | 1.05 (0.80–1.37) | 210 | 235 | 0.97 (0.78–1.20) |
| Time in day care before age 2 | | | | | | | | | |
| No | 1399 | 1489 | 1.00 | 498 | 532 | 1.00 | 901 | 957 | 1.00 |
| < 3 months | 63 | 54 | 1.23 (0.84–1.80) | 22 | 16 | 1.43 (0.73–2.79) | 41 | 38 | 1.12 (0.70–1.79) |
| 3–6 months | 51 | 40 | 1.30 (0.85–1.99) | 25 | 11 | 2.33 (1.08–5.01) | 26 | 29 | 0.96 (0.56–1.65) |
| 7–12 months | 71 | 95 | 0.87 (0.63–1.20) | 25 | 51 | 0.61 (0.37–1.00) | 46 | 44 | 1.17 (0.76–1.79) |
| > 12 months | 160 | 201 | 0.91 (0.73–1.15) | 63 | 77 | 1.09 (0.76–1.57) | 97 | 124 | 0.83 (0.62–1.13) |
| Test for trend | | | *P* = 0.461 | | | *P* = 0.949 | | | *P* = 0.458 |

[a]Excluding day care within 1 year before the diagnostic date (case) or reference date (control). Excluding subjects who were less than 1-year-old at diagnostic date (case) or reference date (control). Odds ratios were derived from conditional logistic model adjusted for maternal race, education, and family income.

**Table 3** Siblings and risk of childhood ALL

| | Total ALL | | | 'Common' ALL CD10+, CD19+, Age 2–5 | | | All other ALL | | |
|---|---|---|---|---|---|---|---|---|---|
| | Cases *n* = 1744 | Controls *n* = 1879 | OR (95% CI) | Cases *n* = 633 | Controls *n* = 687 | OR (95% CI) | Cases *n* = 1111 | Controls *n* = 1192 | OR (95% CI) |
| Number of older siblings | | | | | | | | | |
| None | 730 | 813 | 1.00 | 258 | 287 | 1.00 | 472 | 526 | 1.00 |
| 1 | 634 | 659 | 1.08 (0.93–1.26) | 236 | 234 | 1.11 (0.86–1.43) | 398 | 425 | 1.06 (0.87–1.28) |
| ≥2 | 380 | 407 | 1.05 (0.88–1.26) | 139 | 166 | 0.94 (0.70–1.26) | 241 | 241 | 1.13 (0.90–1.41) |
| Test for trend | | | *P* = 0.447 | | | *P* = 0.842 | | | *P* = 0.279 |
| Interval to birth of next older sibling | | | | | | | | | |
| No older sibling | 730 | 813 | 1.00 | 258 | 287 | 1.00 | 472 | 526 | 1.00 |
| ≤5 years | 855 | 911 | 1.05 (0.92–1.21) | 314 | 333 | 1.03 (0.81–1.30) | 541 | 578 | 1.03 (0.89–1.27) |
| > 5 years | 159 | 155 | 1.18 (0.92–1.51) | 61 | 67 | 1.08 (0.72–1.63) | 98 | 88 | 1.23 (0.89–1.69) |
| Test for trend | | | *P* = 0.200 | | | *P* = 0.692 | | | *P* = 0.219 |

Excluding subjects < 1 year old. Odds ratio were derived from conditional logistic model, adjusted for maternal race, education and family income.

95% CI 0.75–1.24). Detailed analysis by immunophenotypic category revealed no additional associations with ORs of 1.00, 0.90, 0.86 and 0.75 for early pre-B ALL, pre-B ALL, B-cell NOS, and T-cell ALL respectively (data not shown).

No significant associations were present for age at start of day care or time in day care, defined as the total number of days that the subject attended day care. Children with 'common' ALL were as likely to have started day care before 6 months of age as were controls. No effect was seen for starting day care later in childhood.

*© 2000 Cancer Research Campaign*

**Table 4** Infection during infancy and risk of childhood ALL*

| | Total ALL | | | 'Common' ALL CD10+, CD19+, Age 2–5 | | | All other ALL | | |
|---|---|---|---|---|---|---|---|---|---|
| | Cases $n = 727$ | Controls $n = 637$ | OR (95% CI) | Cases $n = 299$ | Controls $n = 270$ | OR (95% CI) | Cases $n = 428$ | Controls $n = 367$ | OR (95% CI) |
| Ear infection | | | | | | | | | |
| None | 386 | 302 | 1.00 | 150 | 111 | 1.00 | 236 | 191 | 1.00 |
| Once | 88 | 81 | 0.86 (0.61–1.22) | 37 | 36 | 0.83 (0.49–1.40) | 51 | 45 | 0.92 (0.58–1.45) |
| 2–4 | 161 | 152 | 0.83 (0.63–1.09) | 72 | 76 | 0.65 (0.43–1.00) | 89 | 76 | 0.99 (0.69–1.43) |
| 5 or more | 75 | 83 | 0.71 (0.50–1.01) | 36 | 40 | 0.64 (0.38–1.08) | 39 | 43 | 0.75 (0.46–1.22) |
| Continuous | 17 | 19 | 0.69 (0.35–1.37) | 4 | 7 | 0.47 (0.13–1.67) | 13 | 12 | 0.79 (0.35–1.79) |
| Test for trend | | | $P = 0.026$ | | | $P = 0.016$ | | | $P = 0.326$ |
| Lung infection | | | | | | | | | |
| None | 575 | 523 | 1.00 | 237 | 224 | 1.00 | 338 | 299 | 1.00 |
| Once | 80 | 58 | 1.32 (0.92–1.89) | 33 | 25 | 1.32 (0.76–2.32) | 47 | 33 | 1.33 (0.82–2.14) |
| 2–4 | 56 | 36 | 1.48 (0.95–2.29) | 24 | 14 | 1.79 (0.89–2.58) | 32 | 22 | 1.28 (0.72–2.27) |
| 5 or more | 16 | 19 | 0.70 (0.35–1.40) | 5 | 7 | 0.51 (0.15–1.74) | 11 | 12 | 0.79 (0.34–1.86) |
| Test for trend | | | $P = 0.330$ | | | $P = 0.403$ | | | $P = 0.595$ |
| Vomiting or diarrhoea | | | | | | | | | |
| None | 545 | 192 | 1.00 | 222 | 213 | 1.00 | 323 | 279 | 1.00 |
| Once | 85 | 62 | 1.26 (0.89–1.80) | 31 | 30 | 1.06 (0.62–1.84) | 54 | 32 | 1.55 (0.96–2.49) |
| 2 or more | 97 | 83 | 1.07 (0.78–1.48) | 46 | 27 | 1.57 (0.93–2.66) | 51 | 56 | 0.78 (0.51–1.19) |
| Test for trend | | | $P = 0.428$ | | | $P = 0.108$ | | | $P = 0.622$ |
| Pressure equalization (PE) tubes | | | | | | | | | |
| No | 649 | 565 | 1.00 | 269 | 241 | 1.00 | 380 | 324 | 1.00 |
| Yes | 77 | 71 | 0.97 (0.69–1.37) | 30 | 29 | 0.94 (0.54–1.63) | 47 | 42 | 0.96 (0.61–1.51) |
| Age at PE tubes | | | | | | | | | |
| No | 649 | 565 | 1.00 | 269 | 241 | 1.00 | 380 | 324 | 1.00 |
| 0–1 years | 30 | 35 | 0.78 (0.47–1.30) | 14 | 15 | 0.86 (0.40–1.85) | 16 | 20 | 0.68 (0.34–1.34) |
| ≥2 years | 47 | 35 | 1.18 (0.75–1.88) | 16 | 14 | 1.01 (0.48–2.15) | 31 | 21 | 1.28 (0.72–2.31) |

[a]Participants in the expanded in-person interview, excluding cases and controls under age of 18 months. Odds ratios derived from unconditional logistic model, adjusted for maternal race, education, age and sex of index child and family income

Examination of day care within strata defined by age at diagnosis of ALL revealed that among children under age 5, any attendance at day care was associated with ALL risks similar to those for children never attending day care (OR 0.90, 95% CI 0.75–1.09); risks were similarly close to unity among children aged 6–10 years (OR –1.00, 95% CI 0.70–1.42), and for children aged 11 and older (OR 1.20, 95% CI 0.78–1.87). Among these age groups, no significant associations were seen between age at starting day care or total number of day care visits and risk of childhood ALL (data not shown). Similar results were obtained when the analysis was restricted to day care attendance prior to 2 or 1 years of age.

### Sibship characteristics (Table 3)

No differences between cases and controls were seen in the number of older siblings. The time interval between the birth of children with ALL and the birth of their next older sibling was similar to that of their controls. No significant difference in sibship size or interval were seen within B-lineage groups (data not shown); however, children with T-lineage ALL were somewhat more likely to have two or more older siblings than were their controls (OR 1.88, 95% CI 1.05–3.36).

### Health history, reported infections (Table 4)

Additional data on the occurrence of infections were available for subjects participating in the in-home interview (see Methods). No differences in reported lung infections or instances of gastroenteritis (vomiting and diarrhoea) were evident for the entire group, among cases of 'common' ALL, or within B-lineage or T-lineage ALL subsets (data not shown). Cases were no more likely to have ever had pressure equalization (PE) tubes placed in the ears (OR 0.97, 95% CI 0.69–1.37). Additionally, no significant differences in ALL risk were found for age at, or occurrence of, tonsillectomy, adenoidectomy, or appendectomy (data not shown).

The risk of ALL was reduced in those who experienced one or more ear infections during the first year of life. Risk of ALL decreased with increasing numbers of reported ear infections during the first year of life with ORs of 0.86, 0.83, 0.71 and 0.69 for 1 episode, 2–4 episodes, 5+ episodes and continuous infections respectively (trend $P = 0.026$). This significant inverse association with ear infections in infancy was most evident in children with 'common' ALL (test of trend $P = 0.016$). Cases and controls were equally as likely to report a history of varicella (OR 1.10, 95% CI 0.84–1.45) among all children with ALL and among 'common' ALL cases and controls (OR 0.96, 95% CI 0.56–1.51).

### Infectous exposures in aggregate

An attempt was made to evaluate infectious exposure in aggregate by the creation of a summary variable including day care data, reported infections and sibship characteristics (Table 5, see footnote of the Table for grouping information). No clear trend was evident when comparing the 'intermediate' or 'most' exposed

**Table 5** Summarized infections exposure level and risk of childhood ALL[a]

| | Total ALL | | | 'Common' ALL CD10+, CD19+, Age 2–5 | | | All other ALL | | |
|---|---|---|---|---|---|---|---|---|---|
| | Cases *n* = 727 | Controls *n* = 637 | OR (95% CI) | Cases *n* = 299 | Controls *n* = 270 | OR (95% CI) | Cases *n* = 428 | Controls *n* = 367 | OR (95% CI) |
| Least exposed group[b] | 110 | 101 | 1.00 | 44 | 36 | 1.00 | 66 | 65 | 1.00 |
| Intermediate exposed group[b] | 519 | 450 | 1.06 (0.78–1.43) | 207 | 193 | 0.87 (0.53–1.42) | 312 | 257 | 1.21 (0.83–1.78) |
| Most exposed group[b] | 98 | 86 | 1.13 (0.76–1.70) | 48 | 41 | 1.07 (0.58–1.99) | 50 | 45 | 1.16 (0.68–2.00) |
| Test for trend | | | $P = 0.541$ | | | $P = 0.790$ | | | $P = 0.511$ |

[a]Participants in the expanded in-person interview, excluding cases and controls under age of 18 months. [b]Least exposed group: no day care prior to age of 2; no older sibling; no ear infection, no lung infection during infancy, no history of PE tubes prior to age of 2. Most exposed group: had at least two of the following conditions: had 7 or more months of day care before age of 2; had an older sibling within 5 years; had five or more ear infections or PE tubes prior to age of 2. Intermediate exposed group: all children who did not fall into least or most exposed groups. Odds ratios were derived from unconditional logistic model, adjusted for maternal race, education, the age and sex of index child and family income groups to the 'least' exposed group for all children with ALL or the subset of children with 'common' ALL.

### DISCUSSION

Three separate, but not exclusive, hypotheses of ALL pathogenesis and infection deserve consideration. Citing the international variations in ALL rates, the development of the ALL 'peak' at different times in different populations, and the higher rates of 'common' childhood ALL among more developed populations, Greaves (1988) has suggested that delaying the occurrence of childhood infections may, in some susceptible children, have an aetiological role in the leukaemogenic process. Kinlen has suggested that childhood ALL occurs as a rare response to a specific viral agent or agents and that excesses of childhood leukaemia are promoted by marked urban–rural population mixing, as a result of an increased level of contacts between susceptible and infected individuals. Studies describing an increased occurrence of ALL in British 'New Towns' and other areas of population mixing support this theory (Kinlen, 1988, 1995; Kinlen et al, 1990; Alexander et al, 1997). Smith (1997) has suggested an aetiological role for a viral agent infecting a susceptible mother during pregnancy. JC virus, a polyoma virus, has been proposed as a candidate virus, but there are no data testing this hypothesis in childhood ALL. As part of a large study of childhood ALL undertaken to evaluate risk factors within biologically defined subgroups, we sought to test the hypothesis that fewer infections during early childhood increase the risk of ALL through the use of surrogate indicators of infection including day care, sibship distribution and size, and maternal reporting of her child's illnesses.

Studies of children attending day care in the USA have shown a higher frequency of upper respiratory infections, otitis media, and gastroenteritis compared to children not attending day care (Haskins and Kotch, 1986; Wald et al, 1991; Hansen, 1993; Reeves et al, 1993). Therefore, we postulated that children with ALL would attend day care less often and/or for shorter duration than controls. Overall, and among children with 'common' ALL, we found no protective effect. Moreover, examination of day care 'exposure' variables, including age at initiation of day care and time in day care revealed no evidence for an association. No 'threshold' of day care exposure (time in day care) was present as was reported for 'creche' attendance in Athens (Petridou et al, 1993).

In our study, mothers of children with 'common' ALL reported fewer ear infections than control mothers. This association was confined to the 'common' ALL group. We observed no differences for reported episodes of colds, lung infections, or gastroenteritis between the cases and controls. The reason for this apparent discrepancy is unclear, although maternal recollection of ear infections may be more accurate than recall of the other infectious diseases investigated, as the diagnosis of otitis media is virtually always made by a physician and usually results in treatment with a course of antibiotics. Infections less likely to result in a visit to the physician or treatment with antibiotics may be less reliably recalled. Nevertheless, it is noteworthy that no difference was noted for the frequency of PE tubes (often placed for recurrent ear infections) for the group overall or any of the leukaemia subsets.

The number of, and interval between, the birth of the index child and older siblings were also investigated as a surrogate of exposure to infection, based on the hypothesis that firstborn children or those children whose siblings are much older are less likely to be exposed to infection and thus at an increased risk of ALL (James, 1990). In contrast to our previous data showing an elevated risk of ALL with longer birth intervals (> 5 years) (Kaye et al, 1991), no evidence of an effect of birth order or interval was evident in this investigation.

Given the breadth of interactions that determine any child's frequency or timing of infections, we attempted to create a summary variable that would define the most and least infection exposed groups. This variable, composed of day care attendance, reported episodes of otitis media in infancy, sibship characteristics and need for PE tubes did not support the hypothesis that the 'most exposed' subset of children may be at decreased risk of ALL. Analysis within the immunophenotypically defined ALL strata and the 'common' ALL subset did not reveal any additional associations.

Overall, the results of this analysis provide little support for a relationship between early childhood infections and the risk of childhood ALL. The data reported for ear infections in infancy suggest a protective effect of early, repeated, non-specific

*© 2000 Cancer Research Campaign*

infection, as most episodes of otitis are initiated by viral upper respiratory infection. The specificity of this finding to the 'common' ALL subset provides additional support for this hypothesis. In contrast, the results of the data reported on attendance at day care and sibship characteristics do not.

It is possible that infections do impact on childhood ALL risk and that the limitations of our study precluded ascertainment of some of these relationships. Among worldwide populations there may be significantly more heterogeneity of childhood infectious exposures than within our study population. Thus, actual differences in occurrence and/or timing of infections may not have been great enough within our population to identify small increases (or decreases) in ALL risk. Another potential limitation is the absence of data on the size of the day care facility the study children attended. Thus, we were unable to stratify by day care size, which may impact on the exposures a child has. Nevertheless, day care visits, hours of attendance and age at first attendance of day care provided little evidence of any effect, either among the group overall or the 'common' ALL subset.

Similar investigations of childhood ALL aetiology currently are underway in the UK and Canada. Results from these studies and other hypothesis-directed investigations combining suspected epidemiological and biological determinants for childhood ALL should hopefully clarify any relationship between infection very early in life and the subsequent risk of childhood ALL.

### ACKNOWLEDGEMENTS

Supported in part by grants CA 48051, CA 07036, and the Children's Cancer Research Fund of the University of Minnesota. Contributing Children's Cancer Group investigators, institutions, and grant numbers are given in the Appendix. Grant support from the Division of Cancer Treatment, National Cancer Institute, National Institutes of Health, Department of Health and Human Services.

### REFERENCES

Alexander FE, Chan LC, Lam TH, Yuen P, Leung NK, Ha SY, Yuen HL, Li CK, Li CK, Lau YL and Greaves MF (1997) Clustering of childhood leukaemia in Hong Kong: association with the childhood peak and common acute lymphoblastic leukaemia with population mixing. *Br J Cancer* **75**: 457–463

Feychting M and Ahlbom A (1993) Magnetic fields and cancer in children residing near Swedish high-voltage power lines. *Am J Epidemiol* **138**: 467–481

Greaves MF and Chan LC (1986) Is spontaneous mutation the major 'Cause' of childhood acute lymphoblastic leukaemia? *Br J Haematol* **64**: 1–13

Greaves MF (1988) Speculations on the cause of childhood acute lymphoblastic leukemia. *Leukemia* **2**: 120

Greaves M (1993) A natural history for pediatric acute leukemia. *Blood* **82**: 1043–1051

Greaves MF (1997) Aetiology of acute leukaemia. *Lancet* **349**: 344–349

Gurney JG, Severson RK, Davis S and Robison LL (1995). Incidence of cancer in children in the United States. *Cancer* **75**: 2186–2195

Hansen BWL (1993) Acute illnesses in children. *Scand J Prim Health Care* **11**: 202–206

Haskins R and Kotch J (1986). Day care and illness: evidence, cost, and public policy. *Pediatrics* **77**: 951–982

Hatch EE, Linet MS, Kleinerman RA, Tarone RE, Severson RK, Hartsock CT, Haines C, Kaune WT, Friedman D, Robison LL and Wacholder S (1988) Association between childhood acute lymphoblastic leukaemia and use of electrical appliances during pregnancy and childhood. *Epidemiology* **9**: 234–245

James WH (1990) Further evidence for the hypothesis that one cause of childhood leukaemia is infection. *Paediatr Perinat Epidemiol* **4**: 113–117

Kaye SA, Robison LL, Smithson WA, Gunderson P, King FL and Neglia JP (1991) Maternal reproductive history and birth characteristics in childhood acute lymphoblastic leukaemia. *Cancer* **68**: 1351–1355

Kersey JH (1997) Fifty years of studies of the biology and therapy of childhood leukemia. *Blood* **90**: 4243–4251

Kinlen LJ (1988) Evidence for an infective cause of childhood leukaemia: comparison of a Scottish New Town with nuclear reprocessing sites in Britain. *Lancet* **ii**: 1323–1327

Kinlen LJ (1995) Epidemiological evidence for an infective basis for childhood leukaemia. *Br J Cancer* **71**: 1–5.

Kinlen LJ, Clarke K and Hudson C (1990). Evidence from population mixing in British New Towns 1946–85 of an infective basis for childhood leukaemia. *Lancet* **336**: 577–582

Kleinerman RA, Linet MS, Hatch EE, Wacholder S, Tarone RE, Severson RK, Kaune WT, Friedman DR, Haines CM, Muirhead CR, Boice JD Jr and Robison LL (1997) Magnetic field exposure assessment in a case–control study of childhood leukemia. *Epidemiology* **8**: 575–583

Linet MS, Hatch EE, Kleinerman RA, Robison LL, Kaune WT, Friedman DR, Severson RK, Haines CM, Hartsock CT, Niwa S, Wacholder S and Tarone RE (1997) Residential exposure to magnetic fields and acute lymphoblastic leukemia in children. *N Engl J Med* **337**: 1–7

Parkin DM, Kramarova E, Draper GJ, Masuyer E, Michaelis J, Neglia J, Qureshi S and Stiller CA (1998). *International Incidence of Childhood Cancer, Vol II.* IARC Scientific Publication Number **144**: IARC: Lyon

Petridou E, Kassimos D, Kalmanti M, Kosmidis H, Haidas S, Flyzani V, Tong D and Trichopoulos D (1993) Age of exposure to infections and risk of childhood leukaemia. *Br Med J* **307**: 774

Pui C (1995) Childhood leukaemias. *N Engl J Med* **332**: 1618–1630

Pui C, Behm FG and Crist WM (1993) Clinical and biologic relevance of immunologic marker studies in childhood acute lymphoblastic leukemia. *Blood* **82**: 343–362

Reves RR, Morrow AL, Bartlett AV, Caruso CJ, Plumb RL, Lu BT and Pickering LK (1993) Child day care increases the risk of clinic visits for acute diarrhea and diarrhea due to rotavirus. *Am J Epidemiol* **137**: 97–107

Robison LL and Daigle AE (1984) Control selection using random digit dialing for cases of childhood cancer. *Am J Epidemiol* **12**: 164–166

Ross JA, Davies SM, Potter JD and Robison LL (1994) Epidemiology of childhood leukemia, with a focus on infants. *Epidemiol Rev* **16**: 243–272

Sandler DP and Ross JA (1997). Epidemiology of acute leukemia in children and adults. *Semin Oncol* **24**: 3–16

Savitz DA, Wachtel H, Barnes FA, John EM and Tvrdik JG (1988) Case-control study of childhood cancer and exposure to 60-Hz magnetic fields. *Am J Epidemiol* **128**: 231–238

Smith M (1997) Considerations on a possible viral etiology for B-precursor acute lymphoblastic leukemia of childhood. *J Immunother* **20**: 89–100

Wald ER, Guerra N and Byers C (1991) Upper respiratory tract infections in young children: duration and frequency of complications. *Pediatrics* **87**: 129–133

### APPENDIX

### Participating principal investigators – children's cancer group

| Institution | Investigators | Grant No. |
|---|---|---|
| Group Operations Center<br>Arcadia, California | WA Bleyer, MD<br>A Khayat, PhD<br>H Sather, PhD<br>M Krailo, PhD<br>J Buckley, MBBS, PhD<br>D Stram, PhD<br>R Sposto, PhD | CA 13539 |

© 2000 Cancer Research Campaign

| Institution | Investigators | Grant No. |
|---|---|---|
| University of Michigan Medical Ctr.<br>Ann Arbor, Michigan | R Hutchinson, MD | CA 02971 |
| University of California Medical Ctr.<br>San Francisco, California | K Matthay, MD | CA 17829 |
| University of Wisconsin Hospital<br>Madison, Wisconsin | D Puccetti, MD | CA 05436 |
| Children's Hospital & Medical Ctr.<br>Seattle, Washington | JR Geyer, MD | CA 10382 |
| Rainbow Babies & Children's Hosp.<br>Cleveland, Ohio | S Shurin, MD | CA 20320 |
| Children's National Medical Ctr.<br>Washington, DC | G Reaman, MD | CA 03888 |
| Children's Hospital of Los Angeles<br>Los Angeles, California | P Gaynon, MD | CA 02649 |
| Children's Hospital of Columbus<br>Columbus, Ohio | F Ruymann, MD | CA 03750 |
| Columbia Presbyterian College of<br>Physicians & Surgeons<br>New York, New York | L Wexler, MD | CA 03526 |
| Children's Hospital of Pittsburgh<br>Pittsburgh, Pennsylvania | AK Ritchey, MD | CA 36015 |
| Vanderbilt University School of<br>Medicine<br>Nashville, Tennessee | J Lukens, MD | CA 26270 |
| Doernbecher Memorial Hospital<br>for Children<br>Portland, Oregon | HS Nicholson, MD | CA 26044 |
| University of Minnesota Health<br>Sciences Center<br>Minneapolis, Minnesota | J Neglia, MD | CA 07306 |
| Children's Hosp. of Philadelphia<br>Philadelphia, Pennsylvania | B Lange, MD | CA 11796 |
| Memorial Sloan-Kettering Cancer<br>Center<br>New York, New York | P Steinherz, MD | CA 42764 |
| James Whitcomb Riley Hospital<br>for Children<br>Indianapolis, Indiana | P Breitfeld, MD | CA 13809 |
| University of Utah Medical Center<br>Salt Lake City, Utah | W Carroll, MD | CA 10198 |
| Children's Hospital Medical Ctr.<br>Cincinnati, Ohio | R Wells, MD | CA 26126 |
| Harbor/UCLA & Miller Children's<br>Medical Center<br>Torrance/Long Beach, California | J Finklestein, MD | CA 14560 |
| University of California Medical<br>Center (UCLA)<br>Los Angeles, California | S Feig, MD | CA 27678 |
| University of Iowa Hospitals and<br>Clinics<br>Iowa City, Iowa | R Tannous, MD | CA 29314 |
| Childrens Hospital of Denver<br>Denver, Colorado | L Odom, MD | CA 28851 |
| Mayo Clinic and Foundation<br>Rochester, Minnesota | G Gilchrist, MD | CA 28882 |
| Izaak Walton Killam Hospital<br>for Children<br>Halifax, Canada | D Barnard, MD | – |
| University of North Carolina<br>Chapel Hill, North Carolina | S Gold, MD | – |
| Children's Mercy Hospital<br>Kansas City, Missouri | M Hetherington, MD | – |
| Univ. of Nebraska Medical Center<br>Omaha, Nebraska | P Coccia, MD | – |
| Wyler Children's Hospital<br>Chicago, Illinois | J Nachman, MD | – |
| M.D. Anderson Cancer Center<br>Houston, Texas | B Raney, MD | – |
| New York Univ. Medical Center<br>New York, New York | A Rausen, MD | – |
| Childrens Hosp. of Orange County<br>Orange, California | V Shen, MD | – |

 *© 2000 Cancer Research Campaign*
